# The Potential Role of Calcium Channels in Pulmonary Hypertension

**DOI:** 10.1155/bmri/4121036

**Published:** 2026-03-30

**Authors:** Ping Wu, Dongyang Zhong, Xuqing He, Ying Li, Hui Zhang

**Affiliations:** ^1^ College of Veterinary Medicine, South China Agricultural University, Guangzhou, Guangdong, China, scau.edu.cn

**Keywords:** Ca^2+^, calcium channels, pulmonary hypertension, pulmonary vascular remodeling

## Abstract

Pulmonary hypertension (PH) is a severe cardiovascular syndrome characterized by progressively elevated pulmonary arterial pressure and driven pathologically by sustained vasoconstriction and irreversible vascular remodeling. Growing evidence underscores the central role of calcium ion (Ca^2+^) signaling networks in PH pathogenesis, which dynamically regulate vascular smooth muscle cell (VSMC) contraction, proliferation, metabolic reprogramming, and endothelial–mesenchymal transition. This review summarizes recent advances in three key areas: (1) the molecular mechanisms of Ca^2+^ signaling dysregulation in PH, with emphasis on the causal relationship between disrupted calcium homeostasis and pathological vascular remodeling; (2) the multidimensional crosstalk among transmembrane calcium channels—including voltage‐dependent calcium channels (VDCCs), store‐operated calcium channels (SOCCs), receptor‐operated calcium channels (ROCCs), and mechanosensitive channels (MSCs)—and their dynamic roles in driving PH progression through vascular wall cell contraction, proliferation, immune‐inflammatory responses, and related pathways; and (3) calcium channel‐targeted therapeutic strategies, highlighting recent progress in the development of pharmacological agonists and inhibitors for the management of PH.

## 1. Introduction

Pulmonary hypertension (PH) is a cardiovascular syndrome characterized by abnormally elevated pulmonary circulation pressure, pathologically defined as dysfunctional pulmonary vasculature caused by heterogeneous etiological factors. Clinically, PH is classified into five major subtypes based on distinct pathogenic mechanisms [1]. Despite intergroup differences in etiology, persistent pulmonary vasoconstriction and progressive vascular remodeling remain the core pathological hallmarks shared across all subtypes. Under physiological conditions, the pulmonary circulatory system maintains a hemodynamic profile of high compliance, low pressure, and low resistance to ensure efficient gas exchange. However, when genetic predisposition, chronic hypoxia, inflammatory diseases, or other pathogenic factors disrupt this homeostasis, a cascade of pathological responses is triggered: abnormal vasoconstriction of muscular arterioles and endothelial barrier dysfunction, leading to excessive release of vasoconstrictive mediators, pro‐proliferative factors, and inflammatory chemokines [2]. These alterations establish a vicious cycle of vasoconstriction–proliferation–remodeling, ultimately resulting in progressive vascular occlusion. This not only significantly increases right ventricular afterload but may also lead to irreversible right heart failure and poor prognosis.

In the study of PH pathophysiology, calcium ion (Ca^2+^) signaling has emerged as a key regulatory hub. As a critical secondary messenger, Ca^2+^ precisely controls its transmembrane distribution through voltage‐dependent calcium channels (VDCCs), receptor‐operated calcium channels (ROCCs), and store‐operated calcium channels (SOCCs), thereby modulating essential physiological processes such as vascular tone and cellular proliferation and apoptosis. Notably, aberrant expression and function of calcium channel proteins have been consistently demonstrated in the pulmonary vasculature of PH patients from various etiologies [3–5]. This pervasive dysregulation of calcium homeostasis is now recognized as a central pathophysiological mechanism driving PH development and progression, rather than being limited to a specific patient subset. These findings strongly suggest that calcium homeostasis dysregulation may serve as a central mechanistic node in PH development and progression. A deeper understanding of the spatiotemporal regulation of Ca^2+^ signaling networks in PH pathogenesis will not only refine current disease models but also provide a scientific foundation for novel targeted therapeutic strategies.

### 1.1. Pathogenic Mechanisms of PH

**Figure 1:**
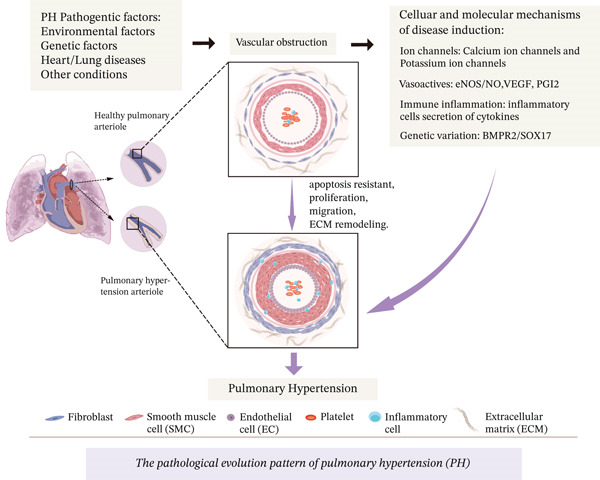
Overview of the pathogenesis of PH. Under the combined influence of genetic predisposition, chronic hypoxia, and cardiovascular diseases, the trilaminar structure of pulmonary vessels (ECs in the intima, SMCs in the media, and fibroblasts in the adventitia) undergoes cascading dysfunction: (1) Ion channel dysregulation (Ca^2+^/K^+^ homeostasis imbalance) induces membrane potential instability; (2) vasoactive substance dysregulation (impaired secretion of eNOS/NO, VEGF, PGI_2_, etc.) leads to vasomotor imbalance; (3) immune‐inflammatory microenvironment (inflammatory cell infiltration) drives chronic vascular injury; and (4) genetic mutations (BMPR2/SOX17 variants) disrupt cellular signal transduction. These pathological mechanisms act in concert to induce an apoptosis‐resistant phenotype in vascular wall cells, triggering aberrant proliferation and migration while promoting excessive extracellular matrix (ECM) deposition, ultimately culminating in irreversible PVR and disease progression in PH. Notes: ECs, endothelial cells; SMCs, smooth muscle cells; eNOS, endothelial nitric oxide synthase; VEGF, vascular endothelial growth factor; NO: nitric oxide; PGI_2_: prostacyclin; BMPR2: bone morphogenetic protein type II receptor. This figure was created by the authors.

The pathogenesis of PH involves complex interactions among multiple pathophysiological processes, with its molecular mechanisms not yet fully elucidated. Based on current evidence, the scientific community has established a core theoretical framework encompassing four major mechanistic categories: imbalance of vasoactive substances, dysregulated immune‐inflammatory responses, genetic susceptibility variants, and ion channel dysfunction (Figure 1). These mechanisms collectively drive the process of pulmonary vascular remodeling (PVR) through synergistic or cascading effects.

#### 1.1.1. Imbalance of Vasoactive Substances

The homeostasis of pulmonary vasculature relies on the dynamic equilibrium of endothelium‐derived vasoactive mediators. Key regulatory molecules include nitric oxide (NO), serotonin (5‐HT), endothelin‐1 (ET‐1), prostacyclin I2/thromboxane A2 system, vasoactive intestinal peptide, and vascular endothelial growth factor (VEGF), etc. Among these, ET‐1 serves as a pleiotropic mediator that induces vasoconstriction, smooth muscle cell (SMC) proliferation, and inflammatory responses through endothelin receptor (ETR) activation [6]. Under physiological conditions, endothelial cells (ECs) maintain vascular tone through precisely regulated synthesis and secretion of these vasoactive substances. However, in pathological states, excessive release of these mediators (particularly the disrupted ET‐1/NO) disrupts vasomotor balance, triggering irreversible vascular remodeling [7]. Furthermore, intracellular Ca^2+^ signaling itself acts as a key regulator of ET‐1 synthesis and secretion in ECs, creating a positive feedback loop that exacerbates vasoconstriction and remodeling [8, 9].

#### 1.1.2. Immune‐Inflammatory Mechanisms

The chronic inflammatory microenvironment serves as a critical driver in the pathological progression of PH. This process is characterized by aberrant perivascular infiltration of immune cells (such as macrophages and T lymphocytes) and sustained overexpression of pro‐inflammatory cytokines (IL‐6, IL‐18, LTB4, etc.) [10–13]. Activated immune cells (particularly macrophages) release diverse inflammatory mediators that damage vascular ECs and increase vascular permeability [14, 15]. Notably, macrophage‐derived IL‐1*β* exerts dual pathological effects by promoting pulmonary artery smooth muscle cells (PASMCs) proliferation through the IL‐1R1/MyD88 signaling pathway while simultaneously exacerbating endothelial dysfunction via suppression of adenylate cyclase activity [16, 17].

#### 1.1.3. Genetic Mechanisms

Genetic predisposition plays a pivotal role in PH pathogenesis, particularly in Group 1 PH. Functionally deleterious mutations in the BMPR2 gene are identified in approximately 70% of familial PH cases and 20% of idiopathic PH patients [18]. These mutations exert pathogenic effects through two distinct mechanisms: (i) potentiation of TGF‐*β*/TAK1/MAPK signaling cascades, resulting in apoptosis resistance and aberrant proliferation of ECs [18, 19] and (ii) disruption of miRNA expression profiles (e.g., dysregulation of miR‐21/145), driving metabolic reprogramming toward a glycolytic phenotype [20, 21]. Furthermore, genome‐wide association studies have progressively identified significant correlations between PH susceptibility and genetic variations in SOX17, BMP10, ALK1, and SMAD8/9 [22], highlighting the extensive genetic heterogeneity underlying this disease.

#### 1.1.4. Ion Channel Mechanisms

The disruption of ion homeostasis in PASMCs represents a pivotal event in PH pathogenesis. Multiple ion channels (K^+^, Cl^−^, Na^+^, Ca^2+^) collectively regulate vascular tone and cellular proliferation by modulating membrane potential and intracellular ion concentrations:

Potassium channels: comprising five major subtypes—voltage‐gated (Kv), ATP‐sensitive, calcium‐activated, inward rectifier, and two‐pore domain potassium channels—their dysfunction induces membrane depolarization. This subsequently activates VDCCs, triggering Ca^2+^ influx and establishing a vicious cycle of depolarization–contraction–proliferation [23–27].

Chloride channels: Channels such as ClC‐3 participate in vascular remodeling by regulating intracellular Cl^−^ concentration, with their aberrant expression being closely associated with hypertensive vascular stiffening [28, 29].

Ca^2+^ signaling network: As the central hub of ion regulation, Ca^2+^ maintains dynamic equilibrium through voltage‐gated/receptor‐operated channels, store‐operated channels, and calcium sensor proteins (like stromal interaction molecule (STIM)/Orai). This intricate system governs critical cellular processes including proliferation, metabolic reprogramming, and epigenetic modifications [30, 31].

Given the paramount importance of calcium channels in PH development, this review will provide a comprehensive analysis of Ca^2+^‐mediated PH regulation, intracellular Ca^2+^ regulatory channels, and extracellular Ca^2+^ regulatory mechanisms.

### 1.2. The Role of Ca^2+^ in PH Pathogenesis

Ca^2+^ is a crucial intracellular second messenger, regulating key physiological processes such as cell membrane stability, contraction–secretion coupling, mitosis, and gene expression through specific concentration oscillations [32–35]. Disruption of its homeostasis can trigger cascading pathological effects and serve as a central driver in the development of PH.

#### 1.2.1. Molecular Basis of Calcium Homeostasis Imbalance

Ca^2+^ overload in PASMCs arises from dysregulation of two major pathways: Intracellular Ca^2+^ release from sarcoplasmic reticulum (SR) stores and extracellular Ca^2+^ influx through plasma membrane channels. SR‐mediated Ca^2+^ efflux is primarily regulated by ryanodine receptors (RyRs) and inositol trisphosphate receptors (IP_3_Rs) embedded in the SR membrane. Their activity is modulated through phosphorylation by regulatory proteins such as FKBP12.6 (calstabin2) and IRAG (IP_3_ receptor–associated cGMP kinase substrate) [36, 37]. The sarc/endoplasmic reticulum Ca^2+^‐ATPase (SERCA), responsible for Ca^2+^ reuptake into SR stores, becomes dysfunctional in PH, leading to progressive SR Ca^2+^ depletion. Extracellular Ca^2+^ enters the cell through channels such as VDCCs, ROCCs, SOCCs, and mechanosensitive ion channels (MSCs). Channel gating is controlled by membrane potential fluctuations or ligand–receptor interactions. Under PH conditions, dysregulated expression of these channels, exemplified by TRPC1/6 upregulation and abnormal glycosylation of Cav1.2 channel *α*‐subunits, induces pathological sustained Ca^2+^ influx [38, 39].

Collectively, these interconnected mechanisms establish a self‐reinforcing cycle of “Ca^2+^ signaling dysregulation–vasoconstriction/remodeling–hemodynamic deterioration”, ultimately driving the progression toward an irreversible PH phenotype.

#### 1.2.2. Ca^2+^ Signaling and Pulmonary Vasoconstriction

Sustained pulmonary vasoconstriction represents a fundamental pathological basis for PH development, with Ca^2+^ playing a central regulatory role. In idiopathic PH, persistent vasoconstriction directly contributes to elevated pulmonary vascular resistance (PVR) and pulmonary artery pressure (PAP) [35]. This process is mediated by aberrant activation of Ca^2+^ signaling in PASMCs: When intracellular Ca^2+^ concentration increases, Ca^2+^ binds to calmodulin (CaM), forming a complex that activates myosin light chain kinase (MLCK). This kinase catalyzes phosphorylation of the myosin regulatory light chain (MLC20), accelerating actin–myosin crossbridge cycling and ultimately inducing vascular contraction [40, 41]. Notably, the synergistic contraction of PASMCs further amplifies pulmonary vascular tone.

#### 1.2.3. Ca^2+^ Signaling and PVR

**Figure 2:**
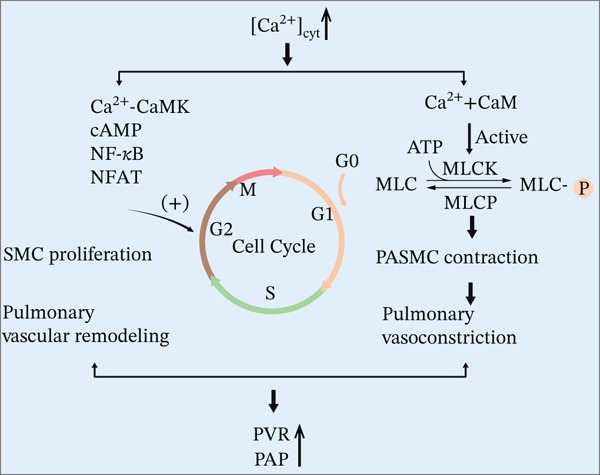
Pathological cascade driven by Ca^2+^ overload in PH. Right (contraction axis): Ca^2+^ binds CaM, activating myosin light chain kinase (MLCK), which phosphorylates myosin light chains (MLC‐P) to drive actomyosin crossbridging and sustained vascular contraction. Membrane depolarization from contraction further activates voltage‐dependent Ca^2+^ channels (VDCCs), creating a positive feedback loop with transient receptor potential canonical (TRPC) channels. Left (proliferation axis): Ca^2+^/CaM activates Ca^2+^/calmodulin‐dependent kinase II (CaMKII), which stimulates the nuclear factor of activated T cells (NFAT) pathway, upregulating cell‐cycle regulators like cyclin D1 to propel cells from G0/G1 into S phase. These combined pathways synergistically increase pulmonary vascular resistance (PVR) and pulmonary artery pressure (PAP), fueling disease progression. *This figure was created by the authors.*

Abnormal proliferation of PASMCs is also recognized as a critical contributor to elevated PVR and PAP. This hyperproliferative phenotype, which forms the pathological basis of vascular remodeling, is tightly regulated by the Ca^2+^/CaMKII signaling axis. Accumulating evidence indicates that Ca^2+^/CaMKII governs cell cycle progression [42, 43]. Elevated intracellular Ca^2+^ levels activate transcription factors such as NF‐*κ*B, NFAT, and CREB, which subsequently upregulate cyclin expression (e.g., cyclin D1/E). This molecular cascade drives G1/S phase transition, thereby establishing Ca^2+^ as a master regulator of cell cycle dynamics in PASMCs (Figure 2) [44–46]. Emerging studies further reveal that inhibition of histone deacetylase 10 triggers aberrant PASMCs proliferation by promoting the nuclear translocation of acetylated NF‐*κ*B, which activates the calcium‐sensing receptor [47].

## 2. Regulation of Calcium Channels in PH Pathogenesis

### 2.1. Regulation of Intracellular Calcium Channels in PH Pathogenesis

Intracellular calcium homeostasis is coordinately regulated by the SR and mitochondria. The endoplasmic reticulum (ER) mediates Ca^2+^ release through IP_3_Rs and RyRs, with Ca^2+^ reuptake executed by the SERCA. Mitochondria, conversely, dynamically buffer intracellular Ca^2+^ fluctuations via transmembrane channel systems, including the mitochondrial calcium uniporter (MCU) and Na^+^/Ca^2+^ exchanger (NCLX). Dysfunction of these regulatory systems is closely associated with PH development and progression.

#### 2.1.1. Mitochondrial Calcium Channels

**Figure 3:**
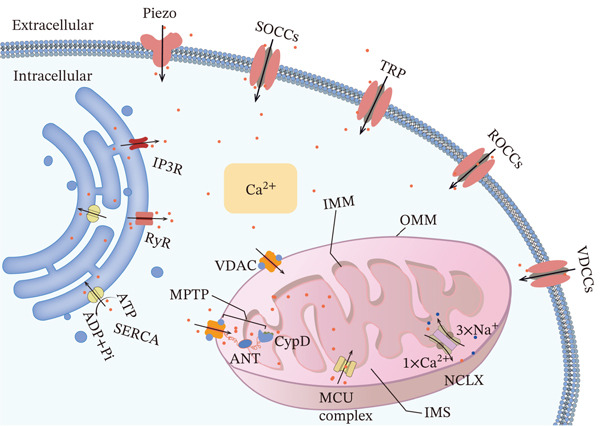
Major pathways for transmembrane and intracellular Ca^2+^ transport. (1) Extracellular influx: Ca^2+^ enters via plasma membrane channels, including VDCCs, ROCCs, SOCCs, TRP channels, and Piezo proteins. (2) SR cycling: Ca^2+^ is released from SR stores via IP_3_Rs and RyRs, and reaccumulated by the SERCA pump. (3) Mitochondrial regulation: Ca^2+^ crosses the OMM via the VDAC and enters the matrix through the MCU on the IMM. Efflux occurs via the NCLX and the MPTP. Notes: ANT, adenine nucleotide translocator; CypD, cyclophilin D; IMM, inner mitochondrial membrane; IMS, mitochondrial intermembrane space; IP_3_R, inositol 1,4,5‐trisphosphate receptor; MCU, mitochondrial calcium uniporter; MPTP, mitochondrial permeability transition pore; NCLX, mitochondrial Na^+^/Ca^2+^ exchanger; OMM, outer mitochondrial membrane; ROCC, receptor‐operated calcium channel; RyR, ryanodine receptor; SERCA, sarco/endoplasmic reticulum Ca^2+^‐ATPase; SOCC, store‐operated calcium channel; SR: sarcoplasmic reticulum; TRP, transient receptor potential; VDAC, voltage‐dependent anion channel; VDCC, voltage‐dependent calcium channel. This figure was created by the authors.

Mitochondria are dynamic organelles that act as a buffer system for cytoplasmic Ca^2+^, enabling rapid absorption of increased cytosolic Ca^2+^. The transport of Ca^2+^ across the mitochondrial outer and inner membranes (OMM and IMM) is mediated by various proteins. These include the voltage‐dependent anion channel (VDAC) in the OMM, the MCU and the NCLX in the IMM, as well as the mitochondrial permeability transition pore (mPTP) spanning the membranes. These channels play critical roles in fundamental mitochondrial physiology (Figure 3).

VDAC, an abundant protein on the OMM, facilitates mitochondrial uptake of Ca^2+^ transferred from other organelles such as the SR and lysosomes, thereby propagating diverse cellular signaling responses [48, 49]. After entering the intermembrane space via VDAC, Ca^2+^ traverses the IMM to reach the mitochondrial matrix. Two primary modes of Ca^2+^ uptake across the IMM have been identified: one mediated by the MCU and the other by a rapid uptake mode (RaM) [50, 51]. RaM is mainly involved in the rapid cellular response to acute stimuli, but the molecular transport mechanism has not been fully defined. MCU transports Ca^2+^ relatively slowly, but its selectivity for Ca^2+^ is high, and it can precisely control the concentration of Ca^2+^ in the mitochondrial matrix to maintain mitochondrial energy metabolism and reactive oxygen species (ROS) production. Additionally, MPTP, a protein channel on the mitochondrial membrane, has been proposed in recent years. Its specific molecular composition is still unclear, but most researchers believe that it is composed of VDAC, ANT, and CypD [52–54]. The opening of MPTP can alter the permeability of the mitochondrial membrane, so as to realize the regulation of mitochondrial Ca^2+^ to regulate pulmonary vasoconstriction [55]. For Ca^2+^ efflux, the mitochondrial NCLX exports Ca^2+^ from the matrix to the cytoplasm by harnessing the electrochemical gradient of Na^+^ and Ca^2+^ across the IMM [56, 57].

Dysregulation of mitochondrial Ca^2+^ homeostasis can therefore influence key cellular processes in vascular cells, such as phenotypic modulation, proliferation, and apoptosis resistance, through mechanisms involving disrupted energy metabolism and altered ROS production [58–60].

#### 2.1.2. ER Ca^2+^ Release Channels

In vascular smooth muscle cells (VSMCs), Ca^2+^ is released from the SR store primarily via two channel systems: the IP_3_R and the RyR. These channels constitute a core source of Ca^2+^ signaling in VSMCs (Figure 3).

The RyR is an intracellular Ca^2+^ release channel located on the SR membrane that enables rapid Ca^2+^ efflux. Three isoforms (RyR1, RyR2, and RyR3) exist with differential tissue distribution; all are present in VSMCs. RyRs contribute to important negative regulatory mechanisms in myogenic tone. Their coupling with calcium‐activated potassium (K_Ca) channels generates spontaneous transient outward currents (STOCs), which directly influence vascular contractile function [61, 62]. Functional studies revealed that knockdown of RyR2 has demonstrated its role in modulating vascular responses to stimuli like hypoxia [63].

The IP_3_R is an intracellular Ca^2+^ release channel that responds to its second messenger inositol 1,4,5‐trisphosphate (IP_3_). Three isoforms (IP_3_R1, IP_3_R2, and IP_3_R3) have been identified. Stimulated by G protein–coupled receptors (GPCRs) on the PM, phospholipase C (PLC) hydrolyzes phosphatidylinositol 4,5‐bisphosphate (PIP_2_) into IP3 and diacylglycerol (DAG). IP_3_ binds and activates IP_3_R, leading to the release of Ca^2+^ stored in the sarcoplasmic/endoplasmic reticulum (SR/ER) into the cytoplasm or adjacent organelles [64]. Concurrently, DAG can act as a lipid messenger to directly or indirectly activate ROCCs on the membrane, inducing extracellular Ca^2+^ influx and initiating Ca^2+^‐dependent cellular responses [65]. Inhibition of IP3R reduces cell proliferation [66], indicating that IP_3_R‐mediated Ca^2+^ signaling plays a pivotal role in regulating VSMCs′ contractility and proliferation.

At present, the function of most Ca^2+^ signaling pathways in mitochondria depends on the formation of contact sites in close connection between mitochondria and SR/ER, which facilitates the direct interaction of ER and other organelles with mitochondria [67–69]. Mitochondria‐associated membranes (MAMs) represent specialized contact sites enabling extensive exchange of information and materials (including Ca^2+^) between mitochondria and ER [70, 71]. Additionally, uncoupling protein 2 (UCP2) on the mitochondrial membrane can transfer ER Ca^2+^ to mitochondria and change mitochondrial function by activating Ca^2+^‐dependent mitochondrial enzymes. Mitochondrial protein 2 (Mfn2), located on the mitochondrial outer membrane, regulates signal transduction (e.g., Ca^2+^ apoptotic signaling) and signal amplification [72, 73].

Cellular stress can disrupt these mitochondrial–ER contact sites. For instance, metabolic inhibition and ER stress have been shown to impair Ca^2+^ shuttling, thereby disrupting the functional unit and contributing to cellular dysfunction [64]. Furthermore, downregulation of mitochondrial fusion proteins like optic atrophy 1 (OPA1) and Mfn2 can exacerbate mitochondrial dysfunction and is associated with adverse tissue remodeling [74]. These findings highlight the critical role of regulated Ca^2+^ transfer between the ER and mitochondria via membrane contact sites in maintaining cellular homeostasis.

#### 2.1.3. The Contribution of Intracellular Ca^2+^Channels in PH

The preceding sections delineate the distinct roles of mitochondrial and SR/ER Ca^2+^ handling machinery. Critically, in the pathogenesis of PH, these intracellular channels do not operate in isolation but form a synergistic and spatially organized network. Their collective contribution lies in establishing and amplifying a state of pathological intracellular Ca^2+^ overload, which in turn fuels sustained vasoconstriction, proliferation, and metabolic reprogramming within the pulmonary vasculature.

Previous studies demonstrated a reduction in mitochondrial Ca^2+^ in PH [75]. Further investigation revealed that this alteration in PH is caused by decreased MICU1 and increased MICU2 expression within the MCU complex, leading to its functional attenuation. Consequently, mitochondrial Ca^2+^ decreases while intracellular Ca^2+^ increases, accompanied by mitochondrial fragmentation and a Warburg effect, collectively promoting PVR [58, 76]. Meanwhile, the decreased function of the MCU complex in PH is attributed to epigenetic mechanisms involving increased expression of miR‐138 and miR‐25, as well as increased ROCK‐mediated Ca^2+^ sensitization in PH, which contributes to sustained vasoconstriction independent of Ca^2+^ influx, further compromising MCU complex function [58, 77]. Furthermore, in PH, inhibition of mitochondrial oxidation leads to mitochondrial membrane hyperpolarization and reduced ROS, thereby raising the threshold for mPTP opening and apoptosis in PASMCs, resulting in enhanced cell survival and proliferation [78, 79]. Additionally, PH impairs endothelial NO synthase activity and downregulates VDAC2 expression in PAECs, potentially further disrupting cellular Ca^2+^ uptake. In summary, metabolic disturbances in mitochondrial Ca^2+^ homeostasis, driven by reduced MCU complex function and mPTP opening, constitute a significant factor promoting PH pathogenesis [80].

Endoplasmic reticulum stress (ERS) is a pivotal factor in the pathogenesis of PH [81]. As the primary intracellular Ca^2+^ store, ERS alters the activity of SR Ca^2+^ channels such as IP_3_Rs and RyRs, causing fluctuations in intracellular Ca^2+^ that further exacerbate PH progression. Studies show that in MCT‐induced PH rat models, inhibition of Mfn2 promotes ERS and upregulates IP_3_R3 expression, facilitating mitochondrial Ca^2+^ uptake [82]. IP_3_R2‐deficient mice develop severe PH upon chronic hypoxia exposure, underscoring the critical contribution of SR Ca^2+^ channels to PH development, compared with WT mice [83]. Interestingly, IP_3_R expression is upregulated in hypoxia‐induced PH rat models, which is potentially linked to different PH‐inducing mechanisms (inflammation vs. hypoxia) [84]. Compared with WT mice, RyR2 knockout mice exposed to chronic hypoxia exhibit a more severe PH phenotype. RyR2 is believed to play a role in the sustained phase of hypoxic pulmonary vasoconstriction [59, 63]. Moreover, RyR2 expression is elevated in PASMCs from hypoxia‐induced PH rats and is thought to participate in regulating TRPV4 function, leading to excessive pulmonary arterial contraction [85]. Regarding SERCA, its expression is reported to be increased in PASMCs from the Milan hypertensive strain rat model, potentially promoting vasoconstriction and elevated blood pressure due to abnormal Ca^2+^ metabolism [86].

In conclusion, the integrated contribution of intracellular calcium channels to PH primarily involves (1) initiating Ca^2+^ release signals through SR/ER channels (IP_3_Rs, RyRs, SERCA), which trigger Ca^2+^‐induced Ca^2+^ release and are subsequently amplified by the activation of plasma membrane channels; and (2) translating Ca^2+^ signals into changes in ATP production, ROS generation, and metabolite availability via mitochondrial channels (MCU, mPTP), thereby supporting the hyperproliferative, apoptosis‐resistant, and Warburg‐effect phenotypes of PH vascular cells. Therefore, targeting intracellular calcium channels—to stabilize ER Ca^2+^ release, inhibit pathological mitochondrial Ca^2+^ uptake, or protect MAM integrity—represents a promising therapeutic strategy aimed at the root of Ca^2+^ dyshomeostasis. This approach may complement methods that block extracellular Ca^2+^ entry.

### 2.2. Extracellular Calcium Channel Dysregulation in PH Pathogenesis

Extracellular calcium influx in PASMCs is predominantly mediated by four transmembrane channel systems, whose dysfunction directly induces pathological Ca^2+^ overload: VDCCs, SOCCs, ROCCs, and MSCs—a novel class of mechanochemical signal transducers including the TRP superfamily and Piezo channels (Figure 3).

#### 2.2.1. Voltage‐Gated Calcium Channels (VDCCs)

VDCCs constitute a family of transmembrane ion channel proteins that modulate diverse cellular physiological processes. These channels exhibit membrane potential‐dependent gating and are composed of four distinct subunits: *α*1, *α*2*δ*, *β*, and *γ* (Table 1). VDCCs exhibit three distinct functional states: resting, activated, and inactivated. At physiological or resting membrane potentials, VDCCs predominantly remain in the closed resting state. When membrane depolarization occurs, VDCCs swiftly transition from the resting to the activated state, enhancing ion permeability, and subsequently undergo rapid inactivation. During membrane repolarization, the ion channels progressively recover from the inactivated state to the resting state [117].

**Table 1:** Classification of VDCCs subunits and subtypes, distribution of expression, and function characteristics.

Subunits	Families	Subtypes	Expression distribution	Function characteristics	References
*α*1	Cav1	Cav1.1	Skeletal muscle	Muscle excitation–contraction coupling	[87]
		Cav1.2	Brain, heart, islets, adrenal glands, and various smooth muscles	Contraction of heart, smooth muscle and blood vessel contraction, regulation of endocrine and cartilage formation, participate in brain learning and memory	[87–89]
		Cav1.3	Brain, heart, inner ear, retina, pancreatic islets, and adrenal glands	Cardiac excitation–contraction coupling, involved in the transmission of auditory and visual signals	[87, 88, 90]
		Cav1.4	Brain and retina	Nerve signal transmission and related to T cell immune response	[87, 91]
	Cav2	Cav2.1	Brain	Release of neurotransmitters	[92, 93]
		Cav2.2	Brain	Transmission of nociceptive signals	[92]
		Cav2.3	Brain, endocrine cells, and various smooth muscles	Associated with a variety of cellular functions, such as insulin secretion and neurotransmitter release	[92, 94]
	Cav3	Cav3.1	Heart, nerve cells, and various smooth muscles	Regulates heart rate, rhythm, and release of neurotransmitters	[95, 96]
		Cav3.2	Brain, nerve cells, and various smooth muscles	Neurotransmitter release, vasoconstriction, and cell proliferation	[97]
		Cav3.3	Spinal cord and sensory neurons	Pain and temperature perception	[98]
*α*2*δ*	*α*2*δ*1		Skeletal muscle, heart, smooth muscle, nervous system, and endocrine tissues	Pain perception and transmission	[99–101]
	*α*2*δ*2		Brain and nervous system	Involved in the regulation of neurotransmitter release and synaptic plasticity	[99, 100]
	*α*2*δ*3		Pain‐related areas in skeletal muscle, heart, brain, and spinal cord	Related to neurotransmitter release and chronic pain state	[99, 100, 102]
	*α*2*δ*4		Retina, endocrine tissue	Related to the occurrence of epilepsy and may be a potential target for some drugs	[100, 103]
*β*	*β*1		Heart, skeletal muscle, and smooth muscle	T cell function regulators and regulate PASMCs	[104–106]
	*β*2		Brain, heart, and lung	Interacts with multiple types of VDCCs and is associated with cardiac hypertrophy	[107, 108]
	*β*3		Brain, spinal cord, and peripheral nervous system	Associated with insulin secretion and temporal lobe epilepsy	[109–111]
	*β*4		Brain	Related to T lymphocyte function	[112, 113]
*γ*			Skeletal and cardiac muscle	Associated with insulin secretion	[114–116]

VDCCs constitute a major pathway for Ca^2+^ influx into PASMCs. Within the cardiovascular system, VDCCs (with Cav3.1 being a key subtype) are expressed in ECs and SMCs, where they modulate intracellular Ca^2+^ levels and associated functions. Patch–clamp studies have identified functional T‐type VDCCs (mainly Cav3.1 and Cav3.2) in PASMCs and established their involvement in both cellular proliferation and contraction. Suppression of T‐type VDCC expression has been shown to effectively halt PH progression [118]. Research using Cav1.2 knockout mice demonstrated attenuated vascular contractility and significantly diminished Ca^2+^ influx induced by 5‐HT or thapsigargin, underscoring the pivotal role of the L‐type channel Cav1.2 in Ca^2+^ signaling pathways [119]. Hypoxic conditions increase the expression of Cav1.2 and Cav3.2 proteins, contributing to pulmonary arterial smooth muscle contraction [120]. Furthermore, miR‐328 can attenuate pulmonary artery vasoconstriction by targeting and reducing Cav1.2 expression, thereby inhibiting Ca^2+^ flux in PASMCs [121]. The auxiliary *β*1 subunit also regulates the hypoxic response in PASMCs, primarily by modulating the constrictive response rather than vascular remodeling [104]. The activity of VDCCs is closely coupled to the membrane potential set by potassium (K^+^) channels. Membrane depolarization, resulting from impaired K^+^ channel function, activates VDCCs, leading to Ca^2+^ elevation. This elevated Ca^2+^ promotes VSMC proliferation and vasoconstriction, which in turn increases intravascular pressure and contributes to vascular remodeling, creating a vicious cycle. Conversely, activation of ATP‐sensitive K^+^ (K_ATP) channels induces membrane hyperpolarization, closing VDCCs, decreasing Ca^2+^ influx, promoting pulmonary vasodilation, and potentially reversing vascular remodeling [122].

#### 2.2.2. SOCCs and ROCCs

SOCE and ROCE represent two main pathways for agonist‐induced extracellular Ca^2+^ influx, both frequently initiated by the activation of GPCRs or receptor tyrosine kinases (RTKs) and the PLC‐mediated hydrolysis of PIP_2_ into DAG and IP_3_ [123, 124]. Despite sharing upstream triggers, they diverge in their core activation mechanisms, temporal dynamics, and downstream consequences (Figure 4).

Figure 4Comparison of SOCE and ROCE Ca^2+^ entry mechanisms. (a) SOCE: Agonist binding to GPCRs or RTKs activates PLC. PLC hydrolyzes PIP_2_ into DAG and IP_3_, and then binds IP_3_R on the SR/ER, releasing stored Ca^2+^ and depleting the SR store. STIM1 senses this depletion, translocates to ER–plasma membrane junctions, and activates Orai1 and TRPC channels, mediating SOCE. (b) ROCE: DAG generated from PIP_2_ hydrolysis acts as a lipid messenger to directly or indirectly activate ROCCs on the plasma membrane, leading to ROCE. ROCE can also secondarily trigger SR Ca^2+^ release via the IP_3_ pathway. The SERCA pump recycles Ca^2+^ back into the SR. Notes: DAG, diacylglycerol; GPCR, G protein‐coupled receptor; IP_3_, inositol 1,4,5‐trisphosphate; IP_3_R, IP_3_ receptor; PIP_2_, phosphatidylinositol 4,5‐bisphosphate; PLC, phospholipase C; ROCE, receptor‐operated calcium entry; ROCC, receptor‐operated calcium channel; RTK, receptor tyrosine kinase; SERCA, sarco/endoplasmic reticulum Ca^2+^‐ATPase; SOCE, store‐operated calcium entry; SOCC, store‐operated calcium channel; SR/ER, sarcoplasmic/endoplasmic reticulum; STIM, stromal interaction molecule; TRPC, transient receptor potential canonical channel. This figure was created by the authors.

**Figure:**
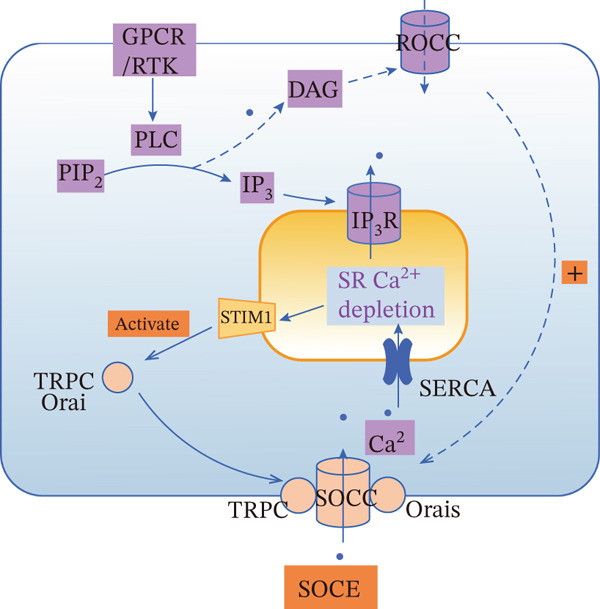
(a) SOCE

**Figure:**
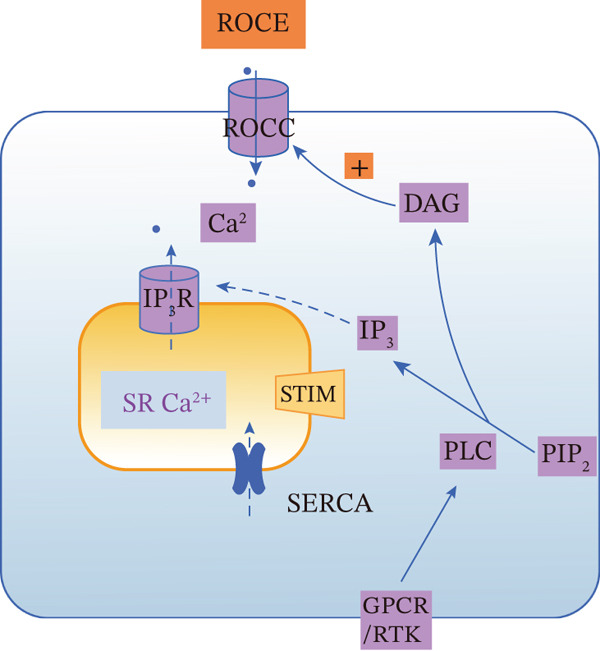
(b) ROCE

SOCE is activated primarily by the depletion of intracellular Ca^2+^ stores within the SR/ER. The decrease in intraluminal Ca^2+^ concentration is sensed by the ER luminal domain of STIM1, a single‐pass transmembrane protein located on the ER membrane [125, 126]. Upon store depletion, STIM1 undergoes oligomerization and translocates to ER–plasma membrane junctions. Here, its cytoplasmic domain physically interacts with and activates the plasma membrane Ca^2+^ channel Orai1, triggering a sustained and capacitative Ca^2+^ influx known as SOCE (Figure 4a) [127]. The TRPC channels, particularly TRPC1, can also be recruited into this complex, forming SOC channels (SOCCs) and modulating the current [128, 129].

SOCE and ROCE are not isolated pathways but exhibit significant cross talk. For instance, TRPC1 can function as a component of both SOCE and ROCE complexes depending on its interaction partners; it forms a SOC channel when complexed with STIM1/Orai1, but can contribute to ROCE when associating with other TRPC isoforms in the absence of STIM1 [128]. This molecular plasticity allows for signal integration and diversification.

In the pathogenesis of PH, both pathways are likely co‐opted to drive the pathological Ca^2+^ overload in PASMCs. ROCE, mediated by vasoconstrictors, may initiate acute increases in Ca^2+^ and contraction. Subsequently, the sustained phase of Ca^2+^ elevation and the proliferation signal may be reinforced and maintained by upregulated SOCE. This cooperative action ensures a persistent pathological Ca^2+^ signal that fuels both vasoconstriction and vascular remodeling. Therefore, therapeutic strategies that simultaneously or sequentially target both entry pathways, or their shared upstream regulators, may offer superior efficacy in disrupting the sustained Ca^2+^ dysregulation that underpins PH progression.

#### 2.2.3. MSCs

MSCs are specialized protein structures that convert physical stimuli (e.g., pressure or deformation of the cell membrane) into electrical and chemical signals, enabling cells to sense environmental changes and respond appropriately [130].

Based on molecular composition and functional characteristics, MSCs can be classified into several major families, including Piezo proteins, TRP superfamily ion channels, degenerin/epithelial sodium channel (DEG/ENaC) family, Kv, and transmembrane channel‐like proteins (TMC). This review focuses on Piezo proteins and the TRP superfamily of ion channels.

##### 2.2.3.1. Transient Receptor Potential (TRP) Ion Channel.

The TRP ion channel family can be divided into two major groups that mediate sensory signaling and can regulate Ca^2+^ homeostasis and influence development. The canonical TRP gene family, one of the TRP subfamilies, comprises seven isoforms (TRPC1‐7) that encode nonselective cation channel proteins. TRPC1, 2, 3, 4, 5, 6, and 7 have been identified in pulmonary vascular smooth muscle from different species, including rats, mice, dogs, and humans. Despite species‐specific expression differences, TRPC is thought to contribute to PH [131]. Researchers observed an approximately 70% increase in Ca^2+^ concentration in hypoxia‐induced PASMCs even with VDCC blockers [132], indicating TRPC proteins′ important role in chronic hypoxia response. This is supported by increased TRPC1 and TRPC6 expression in chronically hypoxic rats′ pulmonary vascular smooth muscle [133] and reduced hypoxic responses in primary‐cultured hPASMCs upon TRPC1 or TRPC6 knockdown [134]. However, TRPC1 and TRPC6 have different mechanisms in hypoxia‐induced PH. TRPC1 mediates PVR in chronic hypoxia [132], whereas TRPC6 mainly mediates pulmonary vasoconstriction in acute hypoxia [135]. Notably, TRPC1/6 dual‐channel inhibition has a synergistic effect on reversing vascular remodeling [136], suggesting the potential for combined targeted therapy.

Transient receptor potential canonical (TRPC) channels play pivotal roles in PH pathogenesis. TRPC1, TRPC3, and TRPC6 are predominantly expressed in PASMCs and have been implicated in PH development [131]. In PH models, TRPC activation regulates PASMC contraction and proliferation. Specifically, TRPC1, TRPC4, and TRPC6 contribute to cellular proliferation, as evidenced by studies showing that knockdown of these channels suppresses PASMC hyperproliferation [38, 137–140]. Furthermore, TRPC channels mediate extracellular matrix (ECM) remodeling in pulmonary arteries by upregulating the expression of type I collagen and fibronectin, thereby promoting vascular wall stiffening [141].

The canonical function of calcium channels dictates that TRPC‐mediated Ca^2+^ influx enhances actin–myosin interactions, leading to increased vasomotor tone and elevated PVR [131]. Notably, TRPC overexpression has also been observed in other cardiovascular inflammatory diseases, including atherosclerosis and arrhythmias [142], suggesting that TRPC may exacerbate PH progression through shared inflammatory pathways. Supporting this hypothesis, Ye et al. demonstrated that treatment with tranilast, a transient receptor potential vanilloid (TRPV) 2 inhibitor, reduces inflammatory cell infiltration and downregulates pro‐inflammatory markers [143]. Recent studies have identified a novel link between fatty acid oxidation and enhanced sensitivity of TRPV4 channels [144]. This finding provides fresh insights into the interplay between Ca^2+^ signaling and metabolic reprogramming in PH, potentially opening new therapeutic avenues targeting energy metabolism dysregulation.

The activation of TRPC is regulated by a variety of factors, including intracellular and extracellular signaling molecules, as well as mechanical stretch activation triggers. In hypoxic cells, the deficiency of O_2_ directly impacts the production of ROS, which in turn regulates the production of various factors, including cytokines, growth factors, chemokines, and transcription factors such as hypoxia‐inducible factor (HIF) in PH [145]. The generated ROS further activate TRPC through redox chemical signaling (e.g., cysteine sulfhydryl reactions) to modulate specific signaling pathways [146, 147]. Although views on the mechanism of TRPC activation vary, it is well established that TRPC channels are activated by membrane receptors coupled to PLC. Thus, TRPC can combine with ROCCs and SOCCs to coregulate Ca^2+^ release. Lin et al.′s study provided evidence that in chronic hypoxia, the use of carotenes as an agonist for the SOCC pathway and DAG as an agonist for the ROCC pathway can upregulate TRPC expression, which can be blocked by TRPC channel blockers La^3+^ and SKF‐96365 [131]. Additionally, TRPC3, TRPC6, and TRPC7 are thought to be involved in ROCE and are sensitive to DAG and GPCRs signaling pathways [148]. SOCC can direct the transfer of TRPC1 to the PM via Orai1‐mediated Ca^2+^, and then STIM1 interacts with TRPC1 to trigger the Gq/PLC–associated signaling pathway and regulate Ca^2+^ concentration [149, 150].

##### 2.2.3.2. Piezo.

Piezo is a member of the MSC family that can be activated by mechanical force changes. Vertebrates have two evolutionarily conserved Piezo proteins: Piezo1 and Piezo2. Much about Piezo′s structure and mechanism remains unknown. Piezo1, a homotrimer forming a propeller‐like transmembrane complex, achieves cation‐selective permeability via a lever‐based mechanotransduction mechanism [151]. It is widely expressed in lung tissues, located in the PM, ER, and mitochondria, and mediates blood flow shear stress (> 10 dyn/cm^2^) and cellular strain (> 15% deformation) signaling [152]. Piezo2, sharing 62% sequence homology with Piezo1, is mainly found in the dorsal root ganglion (DRG) and Merkel cells, mediating touch and proprioception [153, 154], and has excitatory effects on neurons. Notably, there is no direct evidence of Piezo2′s pathological regulation in the PH pulmonary vasculature to date.

Piezo1 senses blood flow shear stress; endothelial Piezo1 detects fluid flow and signals adjacent ECss, causing smooth muscle contraction and raising blood pressure during exercise [155, 156]. As Lhomme et al. reported, Piezo1 induces pulmonary arterial vasoconstriction and diastole by regulating NO production in the pulmonary vascular endothelium [157]. Also, Piezo1 is highly expressed in alveolar epithelial type II cells (AECs). During mechanical ventilation, hyperinflation from mechanical stretch activates Piezo1 in AECs, increasing Ca^2+^ influx [158].

Aberrant activation of Piezo1 drives pulmonary vascular dysfunction through multiple mechanisms, including regulation of cytoplasmic Ca^2+^ levels, participation in cell signaling, influence on vasoconstriction and diastole, and contribution to PVR [159], playing a key role in pulmonary hypertension pathogenesis. Chen et al. noted that Piezo1 is crucial for PASMCs proliferation [160]. Piezo1 is significantly upregulated in PAECs from patients with idiopathic pulmonary hypertension (IPPH) and mice [4]. This upregulation boosts intracellular free Ca^2+^ in PASMCs of IPPH patients, promoting hypercontraction and proliferation [152]. Conversely, Piezo1 deficiency reduces arterial diameter and wall thickness in hypertensive models, indicating its role in vascular remodeling [161]. Thus, Piezo1′s activity in regulating intracellular calcium homeostasis is linked to pathological vasoconstriction and remodeling during PH, playing a key role in inducing cell contraction and proliferation.

#### 2.2.4. The Contribution of Extracellular Calcium Channels in PH

Excessive extracellular Ca^2+^ influx is a central driver of pathological Ca^2+^ overload in PH. The preceding sections of this chapter have detailed VDCCs, SOCCs, ROCCs, and MSCs represented by TRP channels and Piezo channels. Collectively, these extracellular calcium channels do not function in isolation in PH; instead, they form a functionally complementary and signaling‐interwoven network. This network collaboratively induces persistent Ca^2+^ signaling abnormalities in pulmonary vascular cells, thereby driving core pathological phenotypes such as vasoconstriction, abnormal proliferation, and remodeling.

Various extracellular calcium channels mediate Ca^2+^ influx through distinct activation mechanisms, working together to establish and maintain dysregulated intracellular Ca^2+^ homeostasis. VDCCs, as the primary rapid Ca^2+^ influx pathway responding to membrane depolarization, play a crucial role in Ca^2+^ overload associated with PH. In PH patients, membrane potential instability caused by potassium channel dysfunction (for instance, mutations in KCNK3 or KCNA5) and depolarization triggered by vasoconstriction itself can activate VDCCs, initiating Ca^2+^ influx and vasoconstriction [162]. In hypoxia‐induced PH rat models, Ca^2+^ influx dominated by T‐type VDCCs such as Cav3.1 and Cav3.2 induces pulmonary vasoconstriction and PASMCs proliferation [118]. In hypoxia‐induced PH piglet models, abnormal upregulation of L‐type VDCCs is observed, leading to Ca^2+^‐dependent contraction in pulmonary vessels [163]. Furthermore, hypoxia can acutely inhibit Kv channel function and reduce their membrane expression in pulmonary arteries, causing PASMCs depolarization. This mediates the opening of L‐type VDCCs and Ca^2+^ influx, increasing intracellular Ca^2+^ concentration, which subsequently triggers vascular smooth muscle contraction, promotes increased PVR, and ultimately contributes to the development of hypoxic PH [164]. In summary, existing research has linked the upregulation of L‐type VDCC expression in pulmonary arteries to the occurrence of hypoxic PH and suggests that pharmacological inhibition of L‐type VDCC expression during chronic hypoxia may reduce abnormal Ca^2+^‐dependent tension and the development of hypoxic PH. Clinically, L‐type VDCC inhibitors can partially improve symptoms in children with hypoxic PH [165].

The critical role of SOCE in PH is underscored by the upregulation of its molecular components. Increased expression of STIM1, Orai1, and TRPC6 has been documented in PASMCs from PH models and patients, and is essential for enhanced SOCE and associated cellular responses like contraction and proliferation [129, 166]. Genetic or pharmacological inhibition of STIM1 or Orai1 attenuates hypoxia‐induced increases in intracellular Ca^2+^ and ameliorates pathological features in experimental PH [166, 167]. Furthermore, membrane microdomains like caveolae, enriched with the scaffolding protein caveolin‐1, optimize SOCE by facilitating the clustering and interaction of STIM1 and Orai1/TRPC1 [168, 169]. The functional impact of caveolin‐1 appears cell‐context dependent, as its loss in endothelium contributes to dysfunction, whereas its upregulation in PASMCs may exacerbate vascular remodeling [170–172].

In contrast to SOCE, ROCE is activated directly by second messengers (primarily DAG) generated from receptor stimulation, independent of ER Ca^2+^ store depletion. DAG, either directly or through activation of protein kinase C (PKC), gates receptor‐operated Ca^2+^ channels (ROCCs) on the plasma membrane, leading to a more rapid and transient Ca^2+^ influx (Figure 4b). Members of the TRPC subfamily (e.g., TRPC3, TRPC6, and TRPC7) are classical mediators of ROCE [123, 124].

Although direct evidence specifically isolating the role of ROCCs in PH is still evolving, their contribution is evident within the broader context of pathogenic signaling. Elevated levels of vasoactive agonists such as ET‐1 and angiotensin II in PH activate their respective GPCRs, initiating the PLC/DAG pathway and potentially activating ROCCs [173, 174]. This provides a rapid Ca^2+^ signal that can directly contribute to acute vasoconstriction. Furthermore, ROCE can indirectly influence SOCE; the IP_3_ produced concurrently with DAG can release ER Ca^2+^ stores, potentially leading to secondary activation of SOCE, creating a synergistic Ca^2+^ signal.

MSCs like Piezo and some TRP channels directly convert mechanical signals such as hemodynamic stress (pressure, shear stress) and ECM stiffening into Ca^2+^ influx, serving as a critical bridge connecting physical microenvironment changes to pathological Ca^2+^ signaling [175]. Studies report elevated Piezo1 expression in PASMCs, PAECs, and lung tissues of PH patients, as well as in PASMCs from hypoxia‐induced PH mice [4, 152, 176]. This was achieved by regulating pulmonary vascular tension, arterial remodeling, and exacerbating the pathogenesis of PH due to the survival of PAECs. Compared with Piezo1, Piezo2 may function to stabilize cellular Ca^2+^ transport in PH. Loss of Piezo2 function exacerbates the PH phenotype in hypoxia‐induced rat models via the intracellular Ca^2+^/pSRF/VEGFR2 signaling axis, suggesting that enhancing Piezo2 stability and function may help alleviate hypoxia‐induced PH [177]. PASMCs from human PH patients show increased TRPC1 and TRPC3 protein expression [178]. TRPC6 is upregulated in lung and PASMCs from patients with idiopathic PH, accompanied by elevated intracellular Ca^2+^ concentration [38, 179]. Studies on hypoxia‐induced PH animal models also highlight the critical role of TRPC channels in PH pathogenesis. Studies indicate that interventions involving chronic hypoxia, HIF‐1, NOXs, and the NF‐*κ*B pathway upregulate TRPC1 and TRPC6 expression in mouse and rat pulmonary arteries and PASMCs [131, 132, 136, 180]. Among these findings, DAG accumulation induced by hypoxia is a key factor leading to excessive TRPC6 activation in PH mice, resulting in sustained pulmonary vasoconstriction [135, 181]. Pharmacological inhibition or siRNA‐mediated knockdown of these genes can alleviate PH symptoms by reducing PVR and vasoconstriction [132, 182–184]. TRPC3 expression is upregulated in the lungs of monocrotaline‐induced PH rats, and its inhibition alleviates PH manifestations [178]. Combined genetic deletion of TRPC1, TRPC3, and TRPC6 primarily prevents chronic hypoxia‐induced PH by reducing hypoxic pulmonary vasoconstriction rather than modulating vascular remodeling [185]. In chronic hypoxia‐induced PH rat models, TRPM4 and NOX4 are upregulated in pulmonary arteries, and increased ROS contributes to elevated intracellular Ca^2+^[186]. TRPM8 expression is downregulated in pulmonary arteries and PASMCs from chronic hypoxia‐ and monocrotaline‐induced PH rats, and it is thought to be involved in vasorelaxation by affecting Ca^2+^ signaling [187]. Increased TRPV1 expression is found in PASMCs from idiopathic PH patients, leading to increased Ca^2+^ influx and PASMC overproliferation [188]. TRPV4 is upregulated in pulmonary arteries, PASMCs, and PAECs of chronic hypoxia–exposed mice, contributing to increased vasoconstriction, myogenic tone, and pulmonary arterial pressure [85, 189, 190].

In summary, extracellular calcium channels collectively form a functionally complementary signaling network in PH. These channels integrate diverse pathological stimuli, including electrophysiological abnormalities, chemical agonists, and mechanical stress, and convert them into sustained intracellular Ca^2+^ overload. This process directly and synergistically drives excessive pulmonary vasoconstriction and vascular remodeling. The widespread upregulation and gain‐of‐function alterations of these channels solidify their role as core pathogenic effectors. Consequently, targeting this calcium channel network holds significant therapeutic promise, particularly for the development of highly selective modulators and rational combination strategies. Such approaches represent a novel treatment paradigm with the direct goal of correcting the pathological excitability in the final common pathway of PH.

## 3. Challenges, Therapeutic Perspectives, and Future Directions

PH is a multifactorial syndrome sustained by a vicious cycle of vasoconstriction and vascular remodeling. This review synthesizes current evidence positioning dysregulated Ca^2+^ signaling as a central hub in this pathophysiology. Aberrant activity of transmembrane channels (VDCCs, SOCCs, ROCCs, TRP, and Piezo) and intracellular stores (via RyRs/IP_3_Rs), coupled with disrupted mitochondrial–ER cross talk at MAMs, drives pathological Ca^2+^ overload in vascular cells. This orchestrates a coherent pathological program encompassing sustained contraction, hyperproliferation, metabolic reprogramming, and inflammatory activation, ultimately leading to elevated PVR.

### 3.1. Clinical Implications, Translational Barriers and Potential Solutions

Targeting these Ca^2+^‐related signaling axes holds potential therapeutic promise, as evidenced by preclinical efficacy of various channel modulators (Table 2). Ca^2+^ channels (TRPC, Orai1, Piezo1, etc.) serve as more downstream effectors, the direct executors of vasoconstriction and remodeling. Therefore, targeting these channels represents an alternative or combinatory therapeutic strategy aimed at the core executive mechanism of the disease. For instance, patients who respond poorly to existing drugs or those whose characteristic vascular remodeling may be associated with sustained activation of specific channels could find new therapeutic opportunities through channel‐targeted approaches.

**Table 2:** Relevant drug targets.

Drug	Target inhibitor/activator (I/A)	Level of evidence	Main model(s) used	Clinical status/notes	References
Nifedipine	I: VOCC, ROCC	In vivo/in vitro	Mice/human/rat/mesenchymal stem cells/PASMCs	Approved	[191–197]
Verapamil	I: VOCC, ROCC	In vivo/in vitro	Human/rat/PASMCs	Approved	[191, 194, 198–200]
Diltiazem	I: VOCC	In vivo/in vitro	Broiler chicken/dog/human/rat/rabbit/PASMCs	Approved	[201–207]
*ω*‐conotoxin GVIA	I: VOCC	In vivo/in vitro	Dog/mice/rat/rabbit	Preclinical	[208–211]
*ω*‐agatoxin IVA	I: VOCC	In vivo/in vitro	Human/rat/VSMCs	Preclinical	[212–214]
Bay K‐8644	A: VOCC	In vivo	Mice/rat	Preclinical	[215–218]
FPL‐64176	A: VOCC	In vivo/in vitro	Rat/sheep/parathyroid cells	Preclinical	[218–220]
SKF‐96365	I: SOCC, TRP	In vivo/in vitro	Rat/sheep/PASMCs	Preclinical	[221–226]
BTP2	I: SOCC, Orais	In vivo/in vitro	Rat/PASMCs	Preclinical	[223, 227, 228]
Thapsigargin	A: SOCC/I: SERCA	In vivo/in vitro	Pig/rat/PAECs/PASMCs	Preclinical	[229–232]
GsMTx4	I: MSC, Piezos	In vivo/in vitro	Rat/PAECs/PASMCs	Preclinical	[4, 160, 232, 233]
Pyr3	I: TRP	In vivo/in vitro	Rat/PASMCs	Preclinical	[178]
RN‐1734	I: TRP	In vivo/in vitro	Mice/PASMCs	Preclinical	[234]
AMTB	I: TRP	In vivo/in vitro	Rat/PASMCs	Preclinical	[187]
Capsaicin	A: TRP	In vivo/in vitro	Human/rat/PASMCs	Approved	[188, 235–238]
Xestospongin C	I: IP_3_R	In vivo/in vitro	Rat/PASMCs	Preclinical	[85]
2‐APB	I: IP_3_R, TRP, SOCC, Orais (in high concentrations)/A: Orais (in low concentrations)	In vivo/in vitro	Broiler chicken/mice/rat/sheep/PASMCs	Preclinical	[179, 184, 186, 225, 239–242]
Dantrolene	I: RyR	In vivo/in vitro	Pig/rat/PASMCs	Approved	[85, 242–245]
Cicletanine	I: RyR	In vivo/in vitro	Human/rat	Approved	[246, 247]
Yoda1	A: Piezos	In vivo/in vitro	Mice/rat/PASMCs/PAECs	Preclinical	[157, 248]

However, the translation of this promise into clinical success faces significant, interrelated challenges: (1) the lack of subtype‐selective agents leading to systemic off target effects; (2) the inaccessibility of key subcellular signaling nanodomains (like MAMs) to conventional drugs; and (3) the phenotypic heterogeneity of PH, which may demand personalized therapeutic approaches. To overcome these barriers and move the field forward, future efforts must converge on several strategic priorities. First, leveraging recent breakthroughs in structural biology (for instance, cryo‐EM structures of TRP, Piezo, and Orai channels) is crucial for the rational design of next‐generation, allosteric, and subtype‐selective modulators. Second, innovative delivery systems, such as nanoparticle‐based formulations or cell‐specific targeting motifs, should be explored to enhance pulmonary selectivity and facilitate access to subcellular microdomains. Third, there is a pressing need to define patient‐specific “Ca^2+^ signaling phenotypes” through biomarker discovery, enabling stratified clinical trials that test combinatorial or sequential targeting of dominant pathways (for instance, TRPC1/6 dual‐inhibition for remodeling‐predominant PH).

### 3.2. Limitations of This Review

Although this review is aimed at providing a comprehensive overview of the role of Ca^2+^ channels in PH, several inherent limitations should be acknowledged. First, as a review, it is possible that some relevant studies, particularly those published in non‐English languages or in less prominent journals, may have been inadvertently omitted, introducing a potential for selection and language bias. Second, the field of Ca^2+^ signaling in PH is vast and rapidly evolving. To maintain focus and clarity, we necessarily limited our scope to the most prominent channels and pathways and thus may not have covered every proposed mechanism or experimental finding in equal depth. Last but not least, the translational and therapeutic discussions are primarily based on preclinical evidence. The direct applicability of these findings to all subtypes of human PH and the challenges of clinical translation are complex and require ongoing investigation. These limitations underscore the interpretative nature of this synthesis and highlight areas where future systematic reviews and meta‐analyses could provide further quantitative insights.

## 4. Conclusion

In summary, this review draws the following key conclusions: (1) Dysregulated Ca^2+^ signaling is established as a central hub in PH pathogenesis, driving the core pathological features of sustained vasoconstriction and irreversible vascular remodeling. (2) This dysregulation results from the coordinated dysfunction of an interconnected network of calcium‐handling proteins, including extracellular influx channels (VDCCs, SOCCs, ROCCs, and MSCs), intracellular release channels (RyRs, IP_3_Rs), and disrupted organelle cross talk at sites like MCU. (3) Compelling functional evidence confirms the causal role of specific channels (such as TRPC6, Orai1, and Piezo1) in PH, validating them as important therapeutic targets. (4) translating this knowledge faces significant challenges, primarily the lack of subtype‐selective drugs and the inaccessibility of pathogenic signaling nanodomains. Consequently, the future therapeutic paradigm must shift from nonselective blockade to the precise modulation of specific calcium signaling nodes. This will require the rational design of allosteric modulators based on advanced structural biology, innovative targeted drug delivery systems, and a precision medicine approach guided by patient‐specific “calcium signaling phenotypes.” Ultimately, this integrated strategy offers a promising route to transform mechanistic insight into improved clinical outcomes for PH patients.

## Author Contributions

The authors state that Ping Wu, Xuqing He, and Dongyang Zhong jointly participated in the writing, preparation, and revision of the original manuscript, and Hui Zhang and Ying Li were responsible for conceiving and putting forward suggestions for writing and revision.

## Funding

This study was supported by the Guangdong Province Natural Science Foundation Project (2024A1515013284).

## Conflicts of Interest

The authors declare no conflicts of interest.

## Data Availability

The data that support the findings of this study are available from the corresponding author upon reasonable request.
